# Functional Outcome Following Proximal Tibial Osteosarcoma Resection and Reconstruction by Modular Endoprosthesis

**DOI:** 10.1245/s10434-022-12788-3

**Published:** 2022-11-27

**Authors:** Walid Atef Ebeid, Mohammad Hassan Abd-Ellatif Hassan

**Affiliations:** 1 Faculty of Medicine, Cairo University, Cairo, Egypt; 2 Department of Orthopaedic Surgery, Faculty of Medicine, New Surgery Building, 4th Floor, Zagazig University Hospitals, Zagazig City, Egypt

## Abstract

**Purpose:**

The proximal tibia is a common location for osteosarcoma. Modular endoprosthesis is a popular reconstructive option, yet it has been associated with many complications. Our study aimed to evaluate the functional outcome and complications of proximal tibial osteosarcoma patients treated by limb salvage using modular endoprosthesis.

**Methods:**

A retrospective study of a prospective database was performed during the period between January 2000 and July 2017. Fifty-five patients with proximal tibial osteosarcoma underwent resection and modular endoprosthetic reconstruction. The functional outcome was evaluated using the Musculoskeletal tumor society scoring system and knee range of motion. Postoperative complications were classified according to Henderson classification; Type 1 (soft tissue failure), Type 2 (aseptic loosening), Type 3 (structural failure), Type 4 (infection) and Type 5 (local tumor progression).

**Results:**

The mean follow-up period was 71.69 ± 49.76 months. The mean musculoskeletal tumor society score was 26.5 ± 2.22; the mean range of motion was 72.63 ± 25.07, and the mean extension lag was 15.09 ± 15.38. Type 1, type 2, type 3, type 4, and type 5 complications occurred in 7.3%, 14.5%, 21.8%, 23.6%, and 5.5%, respectively. Chest metastasis developed in 10 patients (18.2%). The estimated 5-year and 10-year survival rates for the treated patients were 83.6% and 79.9%.

**Conclusions:**

Proximal tibial osteosarcoma reconstruction with a modular endoprosthesis is a reliable treatment option for retaining limb function. Most complications are manageable.

**Supplementary Information:**

The online version contains supplementary material available at 10.1245/s10434-022-12788-3.

Osteosarcoma is a primary malignant bone tumor originating from the malignant precursor of the mesenchymal stem cell.^1^ Treatment of osteosarcoma was only amputation; however, with the advancement of chemotherapy, imaging, and surgical techniques, limb salvage surgery became the standard treatment option.^2^

The psychological benefits of limb-salvage surgery outweigh the risks, and the functional and oncological results are good.^3^ Reconstruction options for the proximal tibia after tumor resection include allografts, autografts, and endoprosthesis.^4^ Allograft reconstruction’s use was limited by its complications.^5^

Endoprosthetic reconstruction provides early weight-bearing and rapid functional recovery,^6^ yet it is associated with complications due to lack of soft tissue coverage and patellar tendon attachment issue.^7^ In addition, long-term complications such as aseptic loosening, prosthesis fracture, and infections are not uncommon.^8^ A medial gastrocnemius rotational flap has been found to improve soft tissue covering and reduce infection risk.^9^

Our study aimed to answer the following questions: (a) what is the functional and oncological outcome of patients with proximal tibial osteosarcoma treated by chemotherapy and limb salvage using modular endoprosthesis?; (b) what are the complications associated with this procedure?; and (c) what is the estimated limb and prosthesis survivorship following this procedure?

## Materials and Methods

**Fig. 1:**
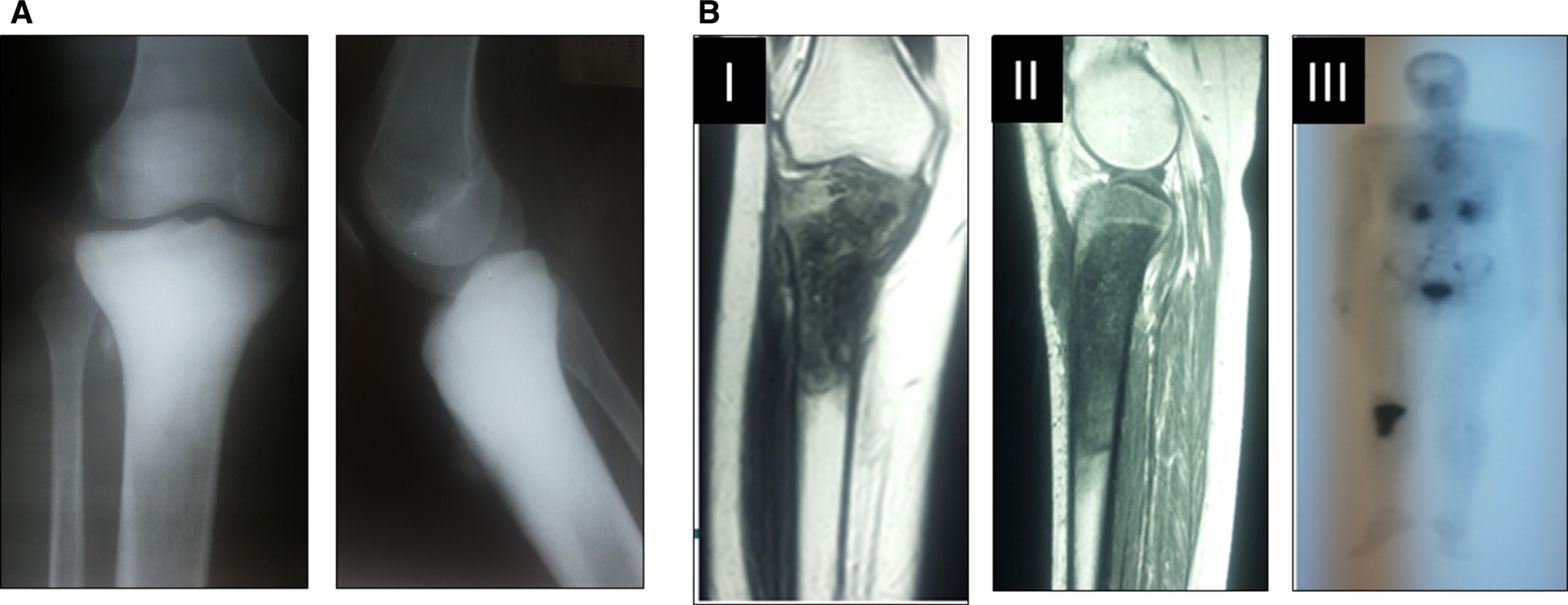
**a** Plain radiograph anteroposterior and lateral view of the knee showing osteoblastic lesion of the proximal tibia. **b** MRI cuts (I) coronal cut (II) sagittal cut showing medullary involvement and soft tissue extension and bone scan (III) showing hot spot

A retrospective analysis of a prospective database was performed at the Center for Preservation and Transplantation of Musculoskeletal tissues at Cairo University. This study was conducted during the period between 2000 and 2017. The modular endoprosthetic replacement was used for reconstruction following resection of proximal tibial osteosarcoma in 55 patients (30 male and 25 female), averaging 19 years old (range 10–51 years). Only one patient aged 10 years received an expandable modular prosthesis. All patients underwent plain radiographs, magnetic resonance (MR) imaging of the knee and tibia, chest computed tomography (CT), and bone scan before undergoing a bone sample for tumor staging (Fig. 1).

Fifty-two patients (94.5%) received neoadjuvant and adjuvant chemotherapy. Three patients with parosteal osteosarcoma (5.5%) did not receive chemotherapy. All patients underwent wide resection of the tumor and reconstruction by modular endoprosthesis. An anteromedial incision was performed, and the neurovascular bundle exposure was done by splitting the soleal arch. Knee arthrotomy was done by cutting the capsule, and the patellar tendon was incised 1 to 2 cm proximal to its tibial tubercle insertion. The cruciate ligaments were transected close to their attachment to the femur. The tibia was cut 2 to 3 cm distal to the most distal marrow involvement. MRI was done preoperatively in all cases and revealed no intra-articular extension, so all cases underwent intra-articular resection. We employed various implants (such as Baumer, Hippokrat, MUTARS, Penta Mears, Egyfix, KOTZ, LINK, Biomechanica) as they changed over time.

**Fig. 2:**
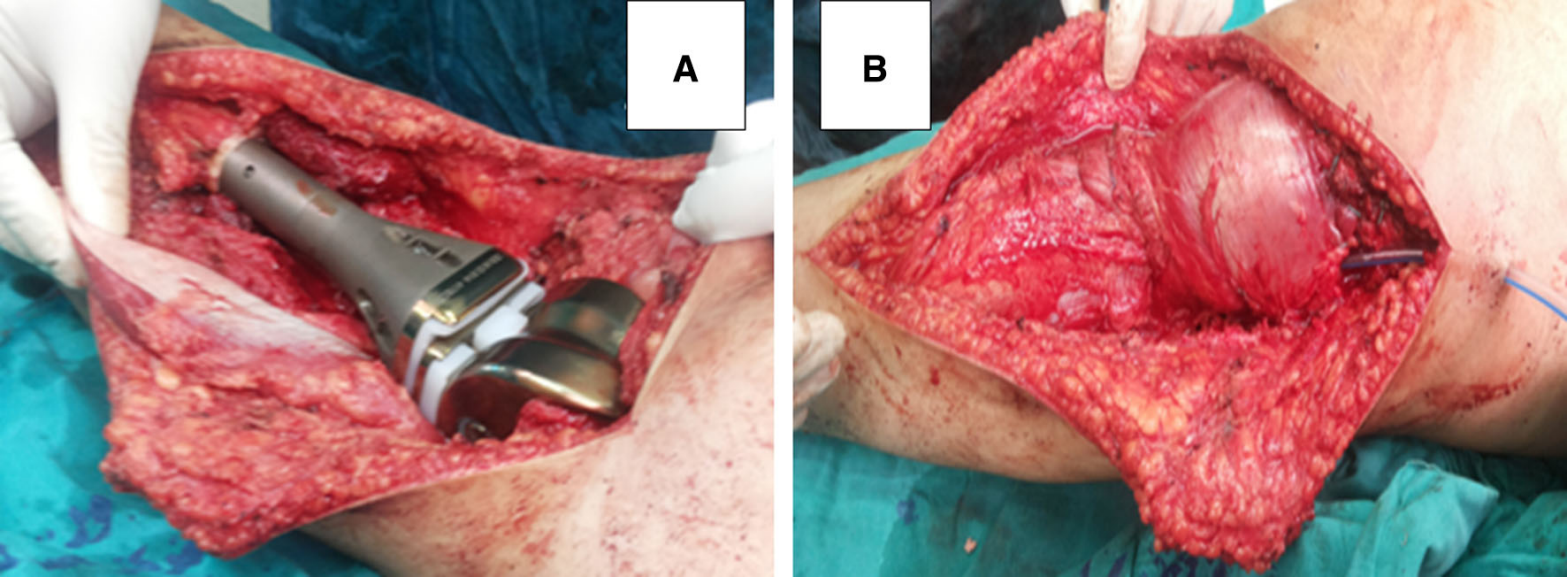
Intraoperative photos showing: **a** Proximal tibial modular prosthesis and development of the medial gastrocnemius flap; **b** Complete prosthetic coverage

The mean length of proximal tibial resection was 15 cm. We used the cemented technique in 36 patients (65.45%) and the non-cemented technique in 19 patients (34.54%). The remaining stump of the patellar tendon was distally advanced and secured to the endoprosthesis with Ethibond, which provided mechanical anchorage. An autologous bone graft was added to the tendon insertion in 10 patients (18.18%). In all patients, the medial gastrocnemius flap was then sewn to the retinacula, patellar tendon, and remaining fascia of the anterior compartment to cover the whole endoprosthesis (Fig. 2). No additional split graft was used in any case.

The limb was kept fully extended using a posterior splint to prevent tension on patellar tendon reconstruction. The splint was removed, and gradual weight-bearing was allowed after six weeks.

In the first year, patients were monitored and evaluated both clinically and radiologically every 6–8 weeks. This interval was doubled with every passing year. Knee range of motion and the MSTS scoring system were used to assess functional outcomes. Failures of the prosthesis were classified using the Henderson classification system.^10^

### Statistical Analysis

All data were collected, tabulated, and statistically analyzed using Statistical Package for the Social Sciences (SPSS) version 24. Continuous variables were checked for normality by using the Shapiro-Wilk test. Continuous Quantitative variables were expressed as the mean ± SD or median (range). While the categorical qualitative variables were expressed as absolute frequencies (number) and relative frequencies (percentage). Mann–Whitney *U* test was used to compare two groups of non-normally distributed variables. Kaplan–Meier (KM) survival analysis estimated free survival rates and overall survival rates. These time-to-event distributions were estimated using the method of the Kaplan–Meier plot and compared using a two-sided exact log-rank test. All tests were two-sided. *P* value < 0.05 was statistically significant, and a *p* valuec≥ < 0.05 was statistically insignificant.

### Ethics

The study protocol was approved by the Ethics Committee of the Faculty of Medicine, Zagazig University (IRB: ZU-IRB #1844-2-3-2015). After explaining the benefits and risks, written informed consent was obtained from the patients or their parents prior to the procedures.

## Results

### Functional Outcome

The median follow-up was 50 (range 27–40) months. The mean MSTS score was 26.5 ± 2.22 (range 18–30), the mean ROM was 72.63 ± 25.07 (range 10–120), and the mean extension lag was 15.09 ± 15.38 (range 0–60). Both the knee range of motion (*p* = 0.002) and resection length (*p* = 0.015) significantly influenced the functional outcome (Table 1).

**Table 1:** Factors affecting functional outcome among osteosarcoma patients (*N* = 55)

	*N*	MSTS II score		Test^a^	*p* value
		Mean ± SD	Median (Min.–Max.)		
All patients	55	26.5 ± 2.2	27 (18–30)	–	–
Sex					
Male	30	26.6 ± 1.9	27 (23–30)	− 0.155	0.877
Female	25	26.4 ± 2.6	27 (18–30)		
Age group (year)					
< 18	33	26.3 ± 2.5	27 (18–30)	− 0.535	0.593
≥ 18	22	26.9 ± 1.6	27 (24–30)		
Length resected (cm)					
≤ 15	31	27.1 ± 1.9	27 (21–30)	− 2.433	**0.015**
> 15	24	25.7 ± 2.4	26.5 (18–30)		
Cement used					
No	19	26.5 ± 2.0	27 (27–29)	− 0.108	0.914
Yes	36	26.5 ± 2.4	27 (27–30)		
Closure					
Uneventful	49	26.5 ± 2.2	27 (18–30)	− 0.096	0.923
Tight	6	26.7 ± 2.3	27 (24–30)		
Operative time (h)					
≤ 4	33	26.9 ± 1.8	27 (23–30)	− 1.289	0.198
> 4	22	25.9 ± 2.6	27 (18–29)		
Chemotherapy					
No	3	25.7 ± 1.5	26 (24–27)	− 1.078	0.281
Yes	52	26.6 ± 2.3	27 (18–30)		
Tumor necrosis					
< 90%	16	26.4 ± 1.8	27 (23–29)	− 0.839	0.401
≥ 90%	36	26.6 ± 2.5	27 (18–30)		
Range of motion					
≥ 80°	25	27.5 ± 1.6	27 (23–30)	− 3.044	**0.002**
< 80°	30	25.7 ± 2.4	26 (18–29)		
Extension lag					
Absent	21	26.8 ± 1.9	27 (23–30)	− 0.663	0.507
Present	34	26.3 ± 2.4	27 (18–30)		
EMR					
Without bone graft	45	26.6 ± 2.2	27 (18–30)	− 0.958	0.338
With graft	10	25.9 ± 2.5	26.50 (21–29)		
Overall complications					
Absent	18	27.2 ± 1.5	27 (24–29)	− 1.483	0.138
Present	37	26.2 ± 2.5	27 (18–30)		
Type 1 complication					
Absent	51	26.7 ± 1.9	27 (21–30)	− 1.588	0.112
Present	4	23.8 ± 4.3	25 (18–27)		
Type 2 complication					
Absent	47	26.4 ± 2.2	27 (18–30)	− 0.780	0.436
Present	8	26.9 ± 2.7	27 (21–30)		
Type 3 complication					
Absent	43	46.4 ± 2.4	27 (18–30)	− 0.437	0.662
Present	12	26.9 ± 1.2	27 (24–29)		
Type 4 complication					
Absent	41	26.7 ± 2.2	27 (18–30)	− 1.134	0.257
Present	14	26.1 ± 2.4	25.50 (23–30)		
Type 5 complication					
Absent	52	26.5 ± 2.3	27 (18–30)	− 0.170	0.865
Present	3	26.3 ± 2.1	27 (24–28)		
Peroneal nerve palsy					
Absent	50	26.4 ± 2.3	27 (18–30)	− 0.657	0.511
Present	5	27.2 ± 0.8	27 (26–28)		

Bold values indicate significant at *p* < 0.05

Continuous variables were expressed as mean ± SD and median (range)

^a^Mann-Whitney *U* test

### Oncological Results

#### Local Recurrence

Local recurrence occurred in three patients (5.5%). Wide resection of the recurrence was done in one patient who is now free of disease, while above-knee amputation was done for the remaining two patients (one patient was lost to follow-up after amputation, and one patient developed stump recurrence). Wide resection was done, and the patient is now free of disease.

#### Chest Metastasis

Ten patients experienced chest metastasis (18.2%); of them, three patients died. Metastectomy was performed for seven patients; of them, two patients survived and were free of disease at the last follow-up. The remaining five patients died of the disease. The assessment of the factors affecting lung metastasis-free survival showed that operative time significantly influenced chest metastasis (*p* = 0.004), as shown in Table 2.

**Table 2:** Factors affecting lung metastasis-free survival (LMFS) among osteosarcoma patients (*N* = 55)

	*N*	Lung metastasis-free survival (LMFS)			Test^a^	*p* value
		Mean 95% CI), months	5-year LMFS	10-year LMFS		
All patients	55	167.1 (146.5–187.7)	79.1%	79.1%	–	–
Sex						
Male	30	175.1 (49.0–201.2)	83.4%	83.4%	0.889	0.346
Female	25	151.1 (121.2–180.9)	74.7%	74.7%		
Age group (year)						
< 18	33	139.7 (115.3–164.1)	75.1%	75.1%	0.582	0.445
≥ 18	22	177.3 (149.4–205.2)	84.8%	84.8%		
Length resected (cm)						
≤ 15	31	178.9 (155.9–201.9)	86.2%	86.2%	1.313	0.252
> 15	24	131.3 (100.2–162.5)	68.8%	68.8%		
Cement used						
No	19	130.4 (109.9–150.9)	86.8%	86.8%	0.848	0.357
Yes	36	160.6 (134.2–186.9)	75.4%	75.4%		
Closure						
Uneventful	49	162.3 (139.4–185.2)	76.4%	76.4%	1.439	0.230
Tight	6	149.0		100%	100%	
Operative time (h)						
≤ 4	33	191.5 (174.6–208.3)	92.9%	92.9%	8.297	**0.004**
> 4	22	118.9 (85.2–152.8)	59.7%	59.7%		
Chemotherapy						
No	3	157.0	100%	100%	0.752	0.386
Yes	52	164.5 (142.7–186.4)	77.7%	77.7%		
Tumor necrosis						
< 90%	16	156.3 (124.4–188.3)	84.4%	84.4%	0.284	0.594
≥ 90%	36	160.5 134.1–186.9)	75.3%	75.3%		
Overall complications						
Absent	18	115.8 (89.8–141.8)	76.2%	76.2%	0.006	0.937
Present	37	167.6 (143.3–191.9)	79.7%	79.7%		
Type 1 complication						
Absent	51	167.3 (145.6–188.9)	79.2%	79.2%	0.065	0.799
Present	4	99.5 (57.9–141.1)	75%	75%		
Type 2 complication						
Absent	47	164.3 141.1–187.5)	77.4%	77.4%	0.234	0.629
Present	8	172.3 (132.4–212.1)	87.5%	87.5%		
Type 3 complication						
Absent	43	158.8 (136.8–180.8)	79.2%	79.2%	0.188	0.665
Present	12	173.4 (135.0–211.8)	81.8%	81.8%		
Type 4 complication						
Absent	41	158.4 (132.3–184.4)	74%	74%	1.512	0.219
Present	14	147.0 (128.1–165.9)	92.9%	92.9%		
Type 5 complication						
Absent	52	168.8 (148.0–189.6)	80%	80%	0.958	0.328
Present	3	70.0 (30.0–110.0)	66.7%	66.7%		

Bold value indicates significant at *p* < 0.05

Categorical variables were expressed as percentage; continuous variables were expressed as mean (95% CI)

^a^Log-rank test

#### Overall Patient Survivorship

The estimated 5-year and 10-year survival rates for the treated patients were 83.6% and 79.9%, respectively (Appendix Fig. 1 in ESM). In our study, we observed that operative time and lung metastasis significantly influenced overall patient survivorship (*p* = 0.002 and *p* < 0.001; Table 3).

**Table 3:** Factors affecting overall survival (OS) among osteosarcoma patients (*N* = 55)

	*N*	Overall survival (OS)			Test^a^	*p* value
		Mean (95% CI), month	5-year OS	10-year OS		
All patients	55	171.6 (150.85–192.22)	83.6%	79.9%	–	–
Sex						
Male	30	187.9 (166.5–209.2)	95.8%	89.4%	3.405	0.065
Female	25	145.4 (111.5–179.4)	68.7%	68.7%		
Age group (year)						
< 18	33	149.5 (127.2–171.8)	80.8%	80.8%	0.202	0.653
≥ 18	22	173.7 (142.6–204.8)	88%	80%		
Length resected (cm)						
≤ 15	31	175.5 (149.6–201.5)	82.5%	82.5%	0.190	0.663
> 15	24	145.7 (118.0–173.4)	85.1%	76.6%		
Cement used						
No	19	129.4 (108.1–150.8)	85.6%	85.6%	0.025	0.874
Yes	36	170.6 (146.6–194.7)	83.6%	79.2%		
Closure						
Uneventful	49	166.3 (142.4–190.1)	81.1%	76.6%	1.348	0.246
Tight	6	149.0		100%	100%	
Operative time (h)						
≤ 4	33	198.6 (188.3–209.0)	97%	97%	9.864	**0.002**
> 4	22	112.2 (72.8–151.6)	61.5%	49.2%		
Chemotherapy						
No	3	157.0		100%	100%	0.628
Yes	52	169.2 (147.1–191.2)	82.4%	78.5%		
Tumor necrosis						
< 90%	16	149.4 (108.0–190.9)	100%	75%	0.000	0.984
≥ 90%	36	170.0 (145.3–194.6)	79.4%	79.4%		
Overall complications						
Absent	18	111.4 (81.5–141.2)	83.9%	67.1%	0.361	0.548
Present	37	176.6 (154.2–198.9)	83.4%	83.4%		
Type 1 complication						
Absent	51	172.1 (150.4–193.8)	84.1%	80.1%	0.322	0.570
Present	4	99.8 (58.6–140.9)	75%	75%		
Type 2 complication						
Absent	47	164.7 (140.1–189.3)	80.1%	75.7%	1.795	0.180
Present	8	194.0		100%	100%	
Type 3 complication						
Absent	43	158.6 (134.6–182.5)	81.7%	76.6%	0.836	0.361
Present	12	188.0 (158.2–217.8)	90%	90%		
Type 4 complication						
Absent	41	165.0 (139.1–191.0)	80.7%	75.6%	0.875	0.350
Present	14	147.8 (130.4–165.2)	92.9%	92.9%		
Type 5 complication						
Absent	52	174.9 (154.9–195.0)	86.2%	82.3%	0.789	0.374
Present	3	75.0 (47.3–102.7)	50%	50%		
Lung metastasis						
Absent		204.0		100%	100%	41.724
Present		51.9 (34.2–69.6)	30%	20%		

Bold value indicates significant at *p* < 0.05

Categorical variables were expressed as percentage; continuous variables were expressed as mean (95%CI)

^a^Log-rank test

### Complications

#### Type 1 (Soft Tissue Failure)

Wound healing issues developed in four patients (7.4%). All of them were poor soft tissue coverage and were treatable. The factors that had significantly influenced type 1 complications were the period in which the surgery was done (before or after 2010) and wound closure issues (*p* = 0.014 and *p* < 0.001), as shown in Table 4.

**Table 4:** Factors affecting type 1 complications among osteosarcoma patients (*N* = 55)

Parameters	Osteosarcoma patients (*N* = 55)		Type 1 complications				Test^a^	*p* value
			Absent (*N* = 51)		Present (*N* = 4)			
	No.	%	No.	%	No.	%		
Year								
< 2010	20	36.4%	16	80%	4	20%	7.549	0.014
≥ 2010	35	63.6%	35	100%	0	0%		
Wound closure								
Uneventful	51	92.7%	51	100%	0	0%	55.000	<0.001
Wound gapping	1	1.8%	0	0%	1	100%		
Inflamed	2	3.6%	0	0%	2	100%		
Slough	1	1.8%	0	0%	1	100%		

Categorical variables were expressed as number (percentage)

^a^Chi-square test

*p* < 0.05 is significant

#### Type 2 (Aseptic Loosening)

Aseptic loosening occurred in eight patients (14.5%). One of them experienced local recurrence, so above-knee amputation was performed, whereas three patients received revision surgery, with their prostheses in good condition till the final follow-up. Four patients refused revision and were lost to follow-up. No factor (age, sex, resection length, operative time, type of fixation, and tumor necrosis) significantly influenced type 2 complications (all *p* > 0.05).

#### Type 3 (Component Breakage and Periprosthetic Fracture)

Two implants (3.6%) failed, one with a damaged tibial stem and the other with a broken bumper and axis screw. They were treated surgically, and the broken component was revised. Nine patients (16.4%) had periprosthetic fractures. Five patients had open reduction and internal fixation by a locked plate, and their fractures were united. Three patients had their fractures united after being treated with an above-knee cast. One patient had a revision with a new prosthesis. No factor (age, sex, resection length, type of fixation, operative time, and tumor necrosis) significantly influenced type 3 complications.

#### Type 4 (Periprosthetic Infection)

Five patients (9.1%) had superficial infections in the early postoperative period. Of them, two patients were treated with antibiotics and improved, while the remaining three patients were treated with debridement and lavage, and their infection was cleared. Deep infection occurred late in eight patients (14.5%). The prosthesis was removed in all eight patients, and a gentamicin-impregnated cement spacer was inserted. One patient had a two-stage revision with another endoprosthesis; three patients had knee arthrodesis by free vascularized fibular graft; two patients had an above-knee amputation, and two were lost to follow-up. No factor (age, sex, resection length, type of fixation, operative time, and tumor necrosis) significantly influenced type 4 complications.

### Limb and prosthesis survivorship

Regarding limb survivorship, the estimated 5-year and 10-year limb survival rates were 88.2% and 88.2%, respectively (Appendix Fig. 2 in ESM). Our study showed that resection length, tumor necrosis, and local recurrence significantly influenced limb survivorship (Table 5). With regards to prosthesis survivorship, the estimated 5-year and 10-year prosthesis survival rates were 82.4% and 62.1%, respectively (Appendix Fig. 3 in ESM). Our study showed that type 2 and 3 complications significantly influenced prosthesis survivorship.

**Table 5:** Factors affecting time to above knee amputation among osteosarcoma patients (*N* = 55)

	*N*	Time to above-knee amputation			Test^a^	*p* value
		Mean (95% CI), month	5-year LS	10-year LS		
All patients	55	182.3 (164.7–200.0)	88.2%	88.2%	–	–
Sex						
Male	30	174.9 (147.8–202.0)	83%	83%	0.524	0.469
Female	25	184.0 (170.7 – 197.4)	96%	96%		
Age group (year)						
< 18	33	167.0 (153.6–180.4)	95%	95%	1.426	0.232
≥ 18	22	170.7 (138.9–202.4)	80.5%	80.5%		
Length resected (cm)						
≤ 15	31	201.0	100%	100%	6.059	**0.014**
> 15	24	137.3 (105.8–168.8)	72.1%	72.1%		
Cement used						
No	19	143.0	100%	100%	1.365	0.243
Yes	36	176.7 (154.5–198.9)	84.7%	84.7%		
Closure						
Uneventful	49	178.7 (157.6–199.8)	85.8%	85.8%	0.768	0.381
Tight	6	146.0		100%	100%	
Operative time (h)						
≤ 4	33	182.0 (161.7–202.4)	88.7%	88.7%	0.075	0.785
> 4	22	154.4 (113.0–195.8)	80%	80%		
Chemotherapy						
No	3	119.3 (59.1–179.6)	66.7%	66.7%	2.003	0.157
Yes	52	186.1 (169.5–202.7)	90.6%	90.6%		
Tumor necrosis						
< 90%	16	131.7 (86.6–176.8)	65.6%	65.6%	8.463	**0.004**
≥ 90%	36	201.0		100%	100%	
Overall complications						
Absent		123.2 (95.5–150.9)	80%	80%	0.005	0.943
Present		183.6 (164.8–202.4)	89.6%	89.6%		
Type 1 complication						
Absent	51	185.4 (168.1–202.7)	89.9%	89.9%	1.653	0.199
Present	4	92.0 (45.6–138.4)	66.7%	66.7%		
Type 2 complication						
Absent	47	181.3 (160.1–202.4)	87.1%	87.1%	0.068	0.794
Present	8	169.3 (129.4–209.1)	87.5%	87.5%		
Type 3 complication						
Absent	43	170.3 (148.3–192.3)	85.5%	85.5%	0.022	0.883
Present	12	185.7 (156.9–214.4)	91.7%	91.7%		
Type 4 complication						
Absent	41	187.9 (169.6–206.2)	91.5%	91.5%	1.125	0.289
Present	14	132.0 (101.1–162.9)	78.8%	78.8%		
Type 5 complication						
Absent	52	189.9 (174.9–204.9)	92.6%	92.6%	20.269	<0.001
Present	3	37.7 (20.7–54.6)	33.3%	33.3%		

Bold values indicate significant at *p* < 0.05

*LS* limb survivors

Categorical variables were expressed as percentage; continuous variables were expressed as mean (95% CI)

^a^Log-rank test

## Discussion

Amputation had been considered the only treatment for high-grade osteosarcoma of the proximal tibia. More than half of the patients died due to metastasis, particularly to the lungs.^2^ The widespread use of neoadjuvant and adjuvant chemotherapy protocols has improved limb salvage safety and patient survival.^11^ The chemotherapeutic combination and limb salvage surgery became the conventional treatment strategy.^2^ Limb salvage employing modular endoprosthetic reconstruction has been shown to be effective in treating proximal tibial osteosarcoma.^6^

The current study included 55 patients with proximal tibial osteosarcoma treated by limb salvage using modular endoprosthesis and followed up for a minimum of 2 years. The functional and oncological outcomes, as well as the factors that influence them, including patient characteristics, tumor features, and operative data, were assessed.

The incidence of local recurrence in the current study was 5.5%. This is comparable to Myers et al.^12^ Their rates of local recurrence varied from 0 to 16%. In the present study, the incidence of chest metastasis was 18.2%. This is consistent with the findings of Mavrogenis et al.^13^ Their rates varied between 0 and 42%.

We observed that the estimated five and 10-year survival rates for treated patients were 83.6% and 79.9%, respectively. Our 5-year overall survival rates were comparable to that reported in previous literature,^13,14^ where rates ranged from 64 to 93%. Our 10-year overall survival rates were comparable as well,^13,14^ where rates ranged from 62 to 87%.

When we looked at the variables that influence local recurrence, none of them had a statistically significant effect. Similarly, in a study by Puchner et al. (2015),^15^ none of the risk variables were statistically significant predictors in the univariate analysis.

Ahlmann et al. showed that the resection margin, poor response to chemotherapy, pathological fracture, and intravascular tumor extension were risk factors for increased recurrence rate.^16^ Only operative time was shown to have a statistically significant association with overall survival and lung metastases in the present study. This could be attributed to the increased vascularity and size of the more aggressive tumor. In their study, Bacci et al. discovered no link between patient survival and factors including gender, age, the extent of resection, or the presence of pathological fracture ^17^. In the current study, the mean MSTS score was 26.5% (88.3%). The MSTS score was ≥ 22 (good-excellent) in 53 patients (96.3%) and was < 22 (fair-poor) in only 2 patients (3.7%). Our results are comparable to the results of Ilyas et al.^18^ Their results ranged from 61 to 90%.

In the study of Pala et al. (2015),^19^ the functional outcome was good or excellent in 97% of the patients, with no difference between the distal femur and the proximal tibia. However, extensor mechanism reconstruction was essential to the extensor lag and ROM. In the current study, the mean ROM and mean extension lag were 72.6 and 15, respectively. We attributed the decreased ROM in our study to the older version of cemented Baumer prosthesis with maximum flexion of ninety degrees. Our findings are in line with the reported results in the literature.^20^

The current study showed no significant correlation between the MSTS functional score and the extension lag. Niimi et al. reported that patients with an extension lag of more than 30 degrees had worse MSTS functional scores than those with an extension lag lower than 30 degrees.^8^ We found that the only factors statistically influencing functional outcomes were the knee range of motion and resection length. Similar to Mavrogenis et al. and Puchner et al.,^13,15^ there was no correlation between the MSTS function and different extensor mechanism reconstructions.

In the current study, the incidence of type 1 failure was 7.3%.; all of them were poor soft tissue coverage and treatable. This was similar to Kinkel et al.^21^ The incidence rate in their study ranged from 2% to 30%. In the study of Hardes et al., the authors found a statistically significant impact of radiotherapy administration and high BMI on the development of wound healing disturbances.^14^ We observed that the timing of the operation was the sole statistically significant predictor of type 1 complications (patients who were operated on before 2010 had a higher incidence). This was due to the improvement in the learning curve in resection techniques and better soft tissue handling. In the study of Puchner et al., 44% of their patients with soft tissue failure also experienced infections.^15^ Type one failure has been connected to higher infection rates and issues with wound healing**.**

Aseptic loosening of proximal tibial endoprosthesis was one of the most common causes of failures.^22^ In the current study, aseptic loosening occurred in eight patients (14.5%). This incidence was comparable with that in the literature,^13,23^ where rates ranged from 6% to 24%. In the current study, no factors significantly impacted the incidence of aseptic loosening. However, the younger age of the patients, greater length of resection, and smaller diameter of the prosthetic stem were reported as the risk factors for developing aseptic loosening.^22^ In Unwin et al.,^24^ aseptic loosening developed with larger resections. Cementless endoprosthesis was expected to develop bone ingrowth as well as long-term prosthetic stability. However, our results showed no significant difference between cemented and cementless endoprosthesis in the incidence of aseptic loosening.

In the current study, prosthesis breakage occurred in two patients (3.6%). This was comparable to that reported in the literature,^14,15^ where rates ranged from 0% to 6%. Despite no significant correlation between prosthesis breakage and resection length and stem size, Griffin et al.,^25^ reported that the stem breakage incidence increased when using a smaller stem diameter and larger resection length.

The rate of infection in our study was high (23.6%). Previous literature reported rates ranging from 12 to 24%.^13–15^ The proximal tibia prosthetic infection rate was reported to be much higher than in the distal femur.^13^ In the current study, no factors significantly influenced type 4 complications. Similar to other studies,^14^ we observed no association between infection and other factors such as age, resection length, and previous operation. The effect of bone resection length on infection rate does not align with our findings.^9^

One of the main concerns regarding endoprosthesis is its longevity; the prosthesis does not last for life. Several factors impact their median lifespan. In the current series, the 5-year and 10-year survival rates were 82.4% and 62.1%. The implant survival steadily decreased over time, i.e., almost one-third of the prosthesis required removal within 10 years. However, our results were in line with that reported in the literature, ranging from 40% to 93.8% at five years,^26,27^ and from 30 to 86.4% at 10 years.^25,27^ In our study, patients’ characteristics, tumors’ characteristics, and operative data did not impact prosthesis survivorship. The factors that impacted the complications were type 2 (aseptic loosening) and type 3 (structural failure), which necessitated the exchange or removal of the prosthesis.

Similar to the study of Niimi et al. (2012),^8^ no statistical correlations between prosthetic survival and factors including age, gender, peroneal nerve palsy, and extension lag were observed. Although the infection has been reported to be the most common type of prosthetic failure,^19^ there was no statistically significant impact of infection on prosthesis survivorship in our study. Zeegen et al.^28^ showed a statistically significant effect of type 4 complications (infection) on prosthesis survival, with no statistically significant impact of resection length on prosthesis survival.

Removal of the prosthesis does not always mean amputation. Our study showed that the limb survivorship rate was 88.2% at 5 years and 88.2% at 10 years. Our 5-year and 10-year limb survival rate is comparable to that reported in the literature, where 5-year limb survivorship rates ranged from 78 to 95%,^14,15^ and the 10-year limb survivorship rates ranged from 74.5 to 94.7%.^10,14,21^

In the current study, tumor necrosis, resection length, type 5 complications, and wound closure issues impacted limb survivorship. Patients with poor tumor necrosis had local recurrence and eventually amputation. Larger resection denotes larger tumor size and more aggression, and eventually local recurrence and amputation. Myers et al. have documented that local recurrence increases the risk of amputation.^12^ In our study, the amputation incidence increased in larger resections. We observed that the resection length significantly influenced above-knee amputation (*p* = 0.031). Müller et al. documented that resection length was related to prosthetic failure.^29^

Our study has numerous strength points. We included a considerable number of patients and performed a long-term follow-up period. Moreover, we reported extensive data on functional and oncological outcomes. The limitations of the current study include that some patients were lost to follow-up, and others had complications but refused to undergo any surgical management. Different types of endoprostheses and different chemotherapy protocols were used over the long period of the study.

## Conclusions

Although limb salvage surgery using endoprosthetic replacement of the proximal tibia is fraught with many surgical complications, it offers a reliable, safe technique for preserving the limb with good limb function and good quality of life.

## Supplementary Information

Below is the link to the electronic supplementary material.

Supplementary file1 (DOCX 148 KB)
