# Supplementary material for: Functional Outcome Following Proximal Tibial Osteosarcoma Resection and Reconstruction by Modular Endoprosthesis
Source: Ann Surg Oncol. 2022 Nov 27;30(3):1914–25. doi: 10.1245/s10434-022-12788-3 (PMC9908643; doi:10.1245/s10434-022-12788-3)
Supplement: Supplementary file 1 — Supplementary file1 (DOCX 148 KB) [file 10434_2022_12788_MOESM1_ESM.docx]

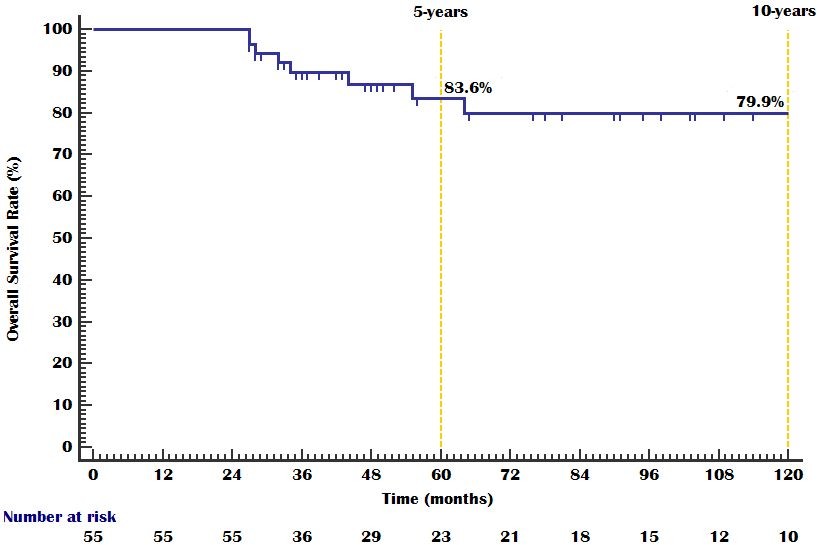


*Appendix Figure 1: Kaplan Meier plot for overall survival rate among the studied osteosarcoma patients (N=55).*


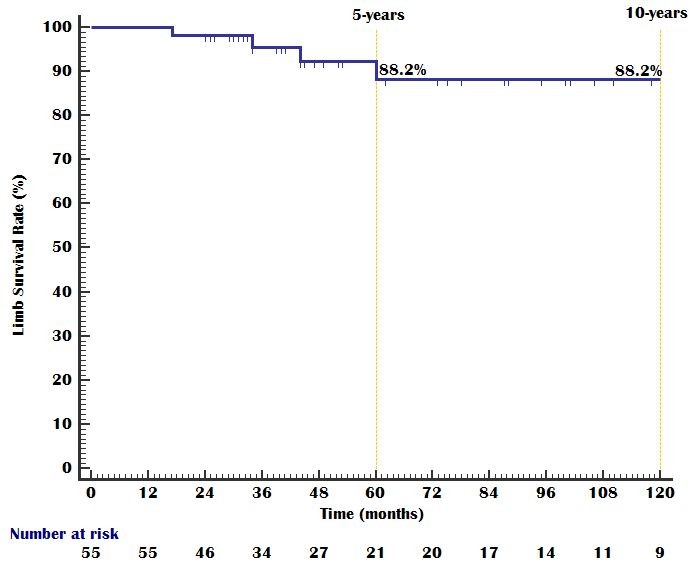


*Appendix Figure 2: Kaplan Meier plot for limb survival rate among the studied osteosarcoma patients (N=55).*


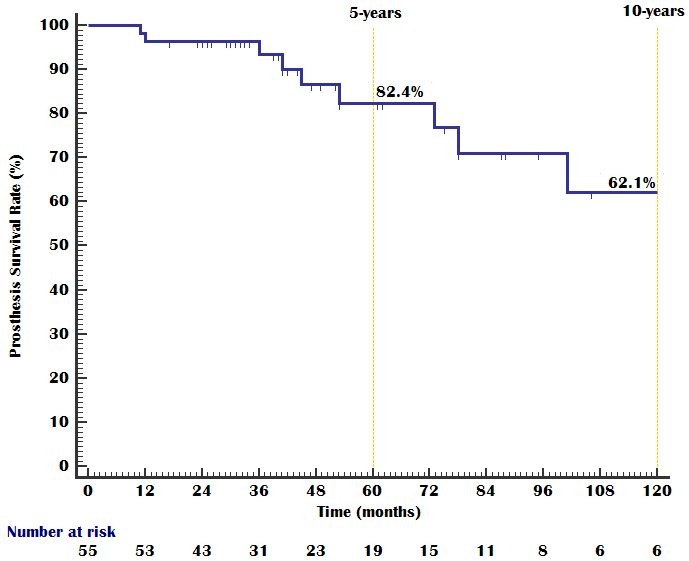


*Appendix Figure 3: Kaplan Meier plot for prosthesis survival rate among the studied osteosarcoma patients (N=55).*
